# Mapping covariance in brain FDG uptake to structural connectivity

**DOI:** 10.1007/s00259-021-05590-y

**Published:** 2021-10-22

**Authors:** Igor Yakushev, Isabelle Ripp, Min Wang, Alex Savio, Michael Schutte, Aldana Lizarraga, Borjana Bogdanovic, Janine Diehl-Schmid, Dennis M. Hedderich, Timo Grimmer, Kuangyu Shi

**Affiliations:** 1 Department of Nuclear Medicine, Klinikum rechts der Isar, School of Medicine, Technical University of Munich, Ismaninger Str. 22, Munich, 81675 Germany; 2 Klinikum rechts der Isar, School of Medicine, Neuroimaging Center (TUM-NIC), Technical University of Munich, Munich, Germany; 3 Shanghai Institute for Advanced Communication and Data Science, Shanghai, China; 4 Department Biology II, Ludwig Maximilian University of Munich, Munich, Germany; 5 Department of Psychiatry and Psychotherapy, Klinikum rechts der Isar, School of Medicine, Technical University of Munich, Munich, Germany; 6 Department of Neuroradiology, Klinikum rechts der Isar, School of Medicine, Technical University of Munich, Munich, Germany; 7 Department of Nuclear Medicine, University Hospital Bern, Bern, Switzerland

**Keywords:** Diffusion tensor imaging, Positron emission tomography, Networks, Tractography, FDG-PET

## Abstract

**Purpose:**

Inter-subject covariance of regional 18F-fluorodeoxyglucose (FDG) PET measures (FDG_cov_) as proxy of brain connectivity has been gaining an increasing acceptance in the community. Yet, it is still unclear to what extent FDG_cov_ is underlied by actual structural connectivity via white matter fiber tracts. In this study, we quantified the degree of spatial overlap between FDG_cov_ and structural connectivity networks.

**Methods:**

We retrospectively analyzed neuroimaging data from 303 subjects, both patients with suspected neurodegenerative disorders and healthy individuals. For each subject, structural magnetic resonance, diffusion tensor imaging, and FDG-PET data were available. The images were spatially normalized to a standard space and segmented into 62 anatomical regions using a probabilistic atlas. Sparse inverse covariance estimation was employed to estimate FDG_cov_. Structural connectivity was measured by streamline tractography through fiber assignment by continuous tracking.

**Results:**

For the whole brain, 55% of detected connections were found to be convergent, i.e., present in both FDG_cov_ and structural networks. This metric for random networks was significantly lower, i.e., 12%. Convergent were 80% of intralobe connections and only 30% of interhemispheric interlobe connections.

**Conclusion:**

Structural connectivity via white matter fiber tracts is a relevant substrate of FDG_cov_, underlying around a half of connections at the whole brain level. Short-range white matter tracts appear to be a major substrate of intralobe FDG_cov_ connections.

**Supplementary Information:**

The online version contains supplementary material available at 10.1007/s00259-021-05590-y.

## Introduction

In the last decade, brain connectivity has evolved as a hot topic of neuroscience. Along with functional magnetic resonance imaging (fMRI), positron emission tomography (PET) with 18F-fluorodeoxyglucose (FDG) represents a valuable tool for exploring neural function in vivo. Of note, FDG-PET can also provide information on brain connectivity. The term metabolic connectivity refers to interrelations between metabolic (FDG) measurements in different brain regions [1]. This approach was shown to yield valuable knowledge on substrates of cognitive reserve [2, 3], working memory [4, 5], impulse control [6], as well as on pathophysiology and diagnosis of neurodegenerative [7–10] and non-neurodegenerative disorders [11, 12]. However, it is still unclear how MC is related to actual structural connectivity, i.e., connectivity through white matter fiber tracts. The latter can be measured in the living human brain using diffusion tensor imaging (DTI). Notably, a few DTI studies have investigated structural substrates of functional connectivity from fMRI data. Measures of structural and functional connectivity were found to be interrelated, and structurally connected cortical regions exhibited a stronger and more consistent functional connectivity than structurally unconnected regions [13]. The goal of the present study was to quantify the degree of spatial overlap between FDG_cov_ and structural connectivity networks. We now prefer the term FDG_cov_, inter-subject covariance of regional FDG-PET measures, over the term metabolic connectivity to avoid a confusion with connectivity measures from dynamic/functional PET acquisitions [14]. To this end, we analyzed data from a large, heterogeneous pool of 303 subjects, both patients with suspected neurodegenerative disorders and healthy individuals.

## Material and methods

### Subjects

We retrospectively analyzed a database of healthy individuals and subjects who were referred to our department as part of a diagnostic work-up for a suspected neurodegenerative disorder. In total, 303 subjects whose structural MRI (sMRI), DTI, and FDG-PET data were available were included. The cohort consisted mainly of patients with mild cognitive impairment [15] and dementia [16], as well as patients with another or unspecified syndrome diagnosis, and healthy individuals. Healthy subjects were recruited mainly via advertisements in local newspapers. They had no history and symptoms of psychiatric and neurologic disorders, no complaints about cognitive impairment (*n* = 36) or complaints that were not confirmed on neuropsychological testing (*n* = 4). Demographic data of the cohort according to a syndrome diagnosis are summarized in Table 1.

**Table 1:** Demographic characteristics

*Group*	*N*	*Female, %*	*Age*	MMSE*
*Healthy*	*40*	*42.5*	*58.7* ± *11.1*	*n.a*
*MCI*	*125*	*42.4*	*66.6* ± *9.5*	*26.7* ± *1.6*
*Dementia*	*89*	*56.2*	*67.1* ± *9.2*	*20.1* ± *4.9*
*Others*	*49*	*34.7*	*63.7* ± *12.4*	*n.a*
*Total*	*303*	*45.2*	*65.2* ± *10.5*	*–*

Data on age and MMSE are given as mean ± standard deviation. *MCI*, mild cognitive impairment; *MMSE*, mini-mental state examination. *MMSE score was not available in 22 healthy subjects, 3 patients with MCI and in 40 patients with other diagnoses

The study was carried out in accordance with the latest version of the Declaration of Helsinki, after the consent procedures had been approved by the local ethics committee. Written informed consent was obtained from all subjects or their legal representatives.

### Image data acquisition

Imaging data were acquired on a fully integrated Siemens Biograph mMR (Siemens Medical Solutions, Knoxville, USA) PET/MR system [17]. PET data were acquired in list mode over 15 min, 30 min after an intravenous injection of approximately 185 MBq ^18^F-FDG. A Dixon T1 MRI sequence was run in parallel with PET to ensure optimal temporal and regional correspondence between two modalities for later attenuation correction. DTI data were acquired using a fast gradient echo-planar imaging sequence with a TE of 82 ms, a TR of 12,100 ms, and a flip angle of 90°. Per subject, 30 volumes with *b* = 800 s/mm^2^ and distinct diffusion-encoding directions and one volume with *b* = 0 s/mm^2^ were acquired. Images had a field of view of 208 mm with 130 × 130 image matrix and 2 mm slice thickness. A high-resolution structural MRI sequence (T1-weighted MPRAGE) was acquired for anatomical correspondence. PET emission data were corrected for random coincidences, dead time, scatter, and attenuation. Resulting sinograms were reconstructed using a filtered back-projection algorithm (FORE + FBP, Siemens syngo MR B18P) with a 5-mm Hamming filter into 192 × 192 × 128 volumes at a field of view of 450 mm. The voxel size was 3.7 × 3.7 × 2.3 mm^3^.

### Preprocessing of image data

The accuracy of the alignment between MRI and PET images was visually inspected in PMOD (PMOD Technologies LLC, CH). The MPRAGE images were then spatially normalized to the Montreal Neurological Institute space [18] using a simultaneous tissue segmentation/spatial normalization tool in SPM12 (Wellcome Trust Centre for Neuroimaging, UCL, London). Resulting gray matter maps were thresholded with a probability value of 0.5. Transformation matrices were then applied to corresponding DTI and PET images. No smoothing was applied. The spatially normalized PET images were then parcellated into 62 non-overlapping regions (region volume ≥ 1 cm^3^) according to the Hammers atlas [19]. The PET images were corrected for partial-volume effects using a voxel-wise approach [20] implemented in PETPVC (Dept. of Nuclear Medicine, UCL). The regional values were scaled using proportional scaling to the mean value of the whole gray matter (segmented from T1 MRI). Although this approach is not optimal for univariate analyses [21–23], it may provide more stable results in multivariate analyses as those utilized here ([24], own unpublished data). DTI volumes were corrected for eddy currents and head motion using FSL [25]. The general processing of the data was performed using Python programming language with the *nipype* library [26, 27].

### *FDG*_*cov*_* network*

A sparse inverse covariance estimation (SICE) method established in our previous studies [5, 8] was employed to calculate FDG_cov_. The FDG PET data are represented as $\mathbf{\rm X}\in {\mathbb{R}}^{n\times m}$, where *m* denotes the number of anatomical regions and *n* is the number of scanned subjects. Thus, ${\mathbf{x}}_{i}, 1\le i\le n$ is a *m*-dimensional vector for subject *i* and is assumed to follow a multivariate Gaussian distribution ${\mathbf{x}}_{i} \sim \mathcal{N}({\varvec{\upmu}},{\varvec{\Sigma}})$ [8], where ${\varvec{\upmu}}\in {\mathbb{R}}^{m}$ is a mean vector, and ${\varvec{\Sigma}}\in {\mathbb{R}}^{m\times m}$ is an underlying covariance matrix. A sample covariance matrix of the FDG data is $\widehat{{\varvec{\Sigma}}}$, and the inverse covariance matrix is estimated as following:

$$\underset{{\varvec{\Theta}}}{\mathrm{max}}(\mathrm{logdet}\boldsymbol{ }{\varvec{\Theta}}-\mathrm{tr}\widehat{{\varvec{\Sigma}}}{\varvec{\Theta}}-\lambda {\Vert {\varvec{\Theta}}\Vert }_{1})$$

where *λ* is a regularization parameter that regularizes the sparsity, and ${\Vert \Vert }_{1}$ is the *L*_1_ norm. This is a LASSO model [28] which keeps ${\varvec{\Theta}}$ sparse: the regularization parameter *λ* directly controls how many connections will be identified, i.e., how many entries will be nonzero. This estimated sparse inverse covariance matrix ${\varvec{\Theta}}$ is treated as FDG_cov_ network thereafter. Only positive entries in the sparse connectivity pattern were considered as connections, resulting in binary matrices [29].

### Structural connectivity network

Camino [30] was employed to fit the diffusion tensors with logistic regression [31] and to perform streamline tractography through fiber assignment by continuous tracking (FACT, [32]). The algorithm was seeded with all white-matter voxels and stopped when reaching a voxel with a fractional anisotropy (FA) value of less than 0.2 or when the curvature of a tract exceeded 50° over 5 mm [33, 34]. From the tractography results, the mean fractional anisotropy (FA) of a given fiber tract (between the ROIs above, if detected) across subjects was implemented in structural connectivity matrices.

### Network efficiency

Network efficiency (NE) analyses were conducted to define a reasonable number of connections for quantification of the network overlap. NE characterizes information transfer in a network [35]. In particular, local efficiency (*LE*) describes a network’s resistance to failure on a small scale, e.g., when a node is removed. As compared to global efficiency, *LE* is not proportional to the number of connections [36], making it more suitable for the above purpose. The network was modeled as a simple graph of *m* vertices (node) and ${n}_{connected}$ edges (connections). Herewith, a node in the network corresponds to a specific anatomical region. For two network nodes $k$ and $l$, the shortest path length ${d}_{kl}$ is the number of edges on the shortest path. For each node *i*, a subnetwork *G*_*i*_ is defined as a neighborhood subgraph of this node. The efficiency of the subnetwork *G*_*i*_ is defined as average inverse of the shortest path lengths ${d}_{kl}$ in this subnetwork. The local efficiency ${E}_{local}$ then averages the efficiencies across the subnetworks of all nodes:

$$LE\left(G\right)=\frac{1}{m}\sum_{i\in G}\frac{1}{L({G}_{i})(L({G}_{i})-1)}\sum_{k\in {G}_{i}}\sum_{k\ne l\in {G}_{i}}\frac{1}{{d}_{kl}}$$

*LE* is a scaled measure ranging from 0 to 1, with a value of 1 indicating maximum *LE* in the network. Conceptually, a high *LE* represents an effective information transfer within their immediate local communities, enabling effective information processing in the network. The *LE* of a comparable random network was employed as reference. Specifically, random connectivity matrices with the same number of connections and degree of distribution were generated using a random rewiring method [35, 37]. Given that the *LE* from a random matrix increases with the number of connections [38], the genuine *LE* (gLE) was employed to eliminate the influence of random effects. It was defined by subtracting the expected *LE* of the randomly rewired networks (mean of bootstrapping results) from the original *LE* of the corresponding FDG_cov_ and structural networks. Following the theory of signal processing [39], we selected a cutoff value at half maximum of gLE to define a focus window for the number of connections (full-width at half maximum) for further analyses. To assess the stability of results across different sampling populations, 100 bootstrap samples were generated by random resampling with replacement.

### *Overlap between FDG*_*cov*_* and structural networks*

Similar to previous studies, sparsity-based thresholding was employed to restrict the number of connections in both networks [35]. Networks with the same number of connections ${n}_{connected}$ according to the NE analysis were generated for pattern comparison. A threshold at tract-averaged FA values was determined such that only the ${n}_{connected}$ strongest structural connections were left. For FDG_cov_, a scalar regularization parameter *λ* of SICE was chosen such that ${n}_{connected}$ entries were nonzero above the diagonal of the resulting inverse covariance matrix [29]. Diagonal elements, representative of self-connections, were ignored to increase the robustness of the regularization.

A convergence ratio (*CR*) is defined by dividing the number of the pairs which are connected in both networks (${n}_{convergent}$) by the number of present connections ($n_{connected}$),

$$CR=\frac{{n}_{convergent}}{{n}_{connected}}$$

**Fig. 1:**
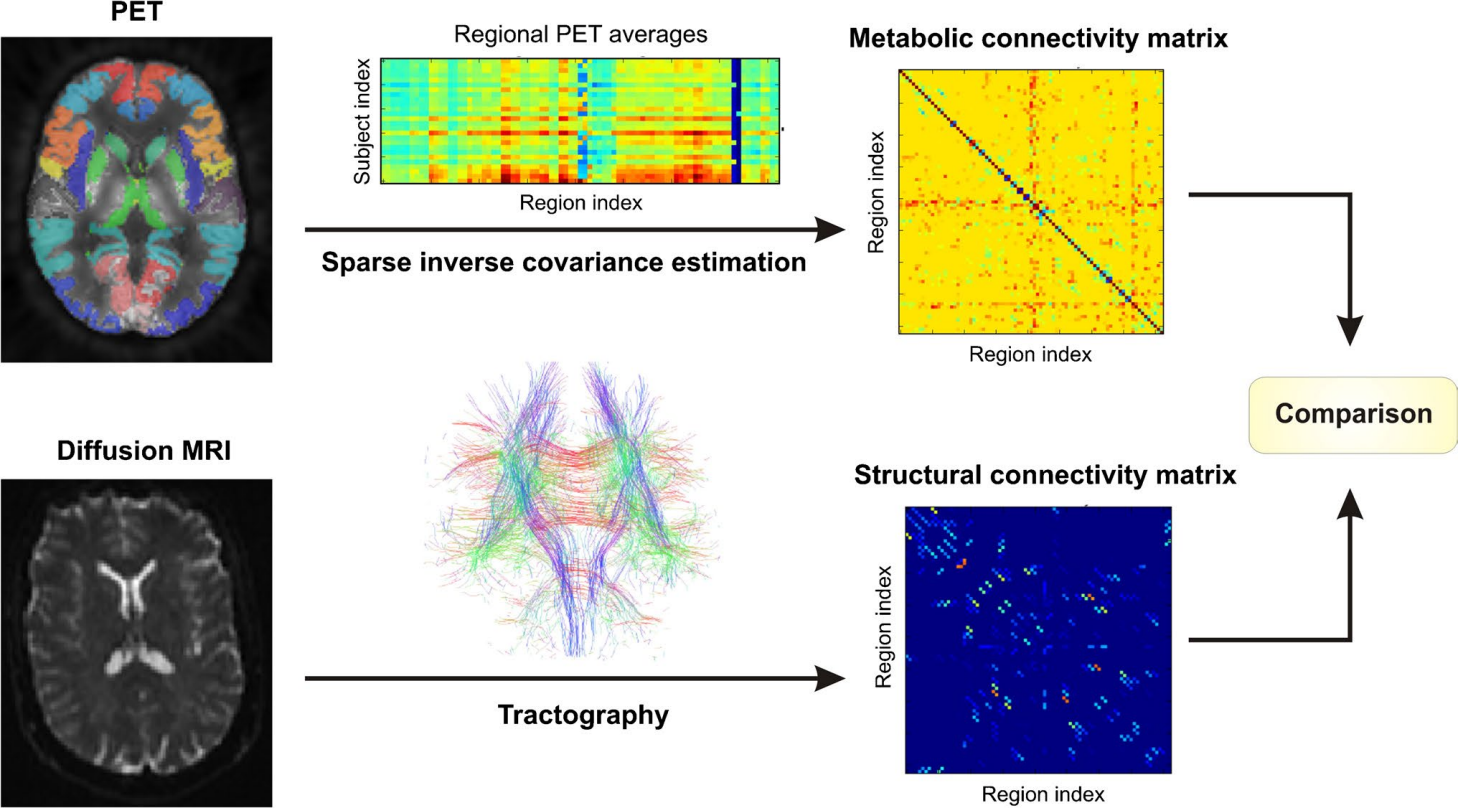
Pipeline of PET and DTI data analyses

This is equivalent to sensitivity in binary classification if the presence of a connection is treated as positive class and is also equivalent to so called Dice similarity coefficient. For individual hemispheres, *CR* was calculated at an unequal number of connections in two networks, and it is adapted to the following expression:

$$CR=\frac{{2n}_{convergent}}{{n}_{metabolic}+{n}_{structural}}$$

where ${n}_{metabolic}$ is the number of functional connections and ${n}_{structural}$ is the number of structural connections. *CR* was analyzed as a function of the number of connections ${n}_{connected}.$ As reference, randomly rewired matrices of the FDG_cov_ and structural networks with the same number of connections were generated 100 times. Figure 1 summarizes the pipeline of the PET and DTI image analyses.

## Results

**Fig. 2:**
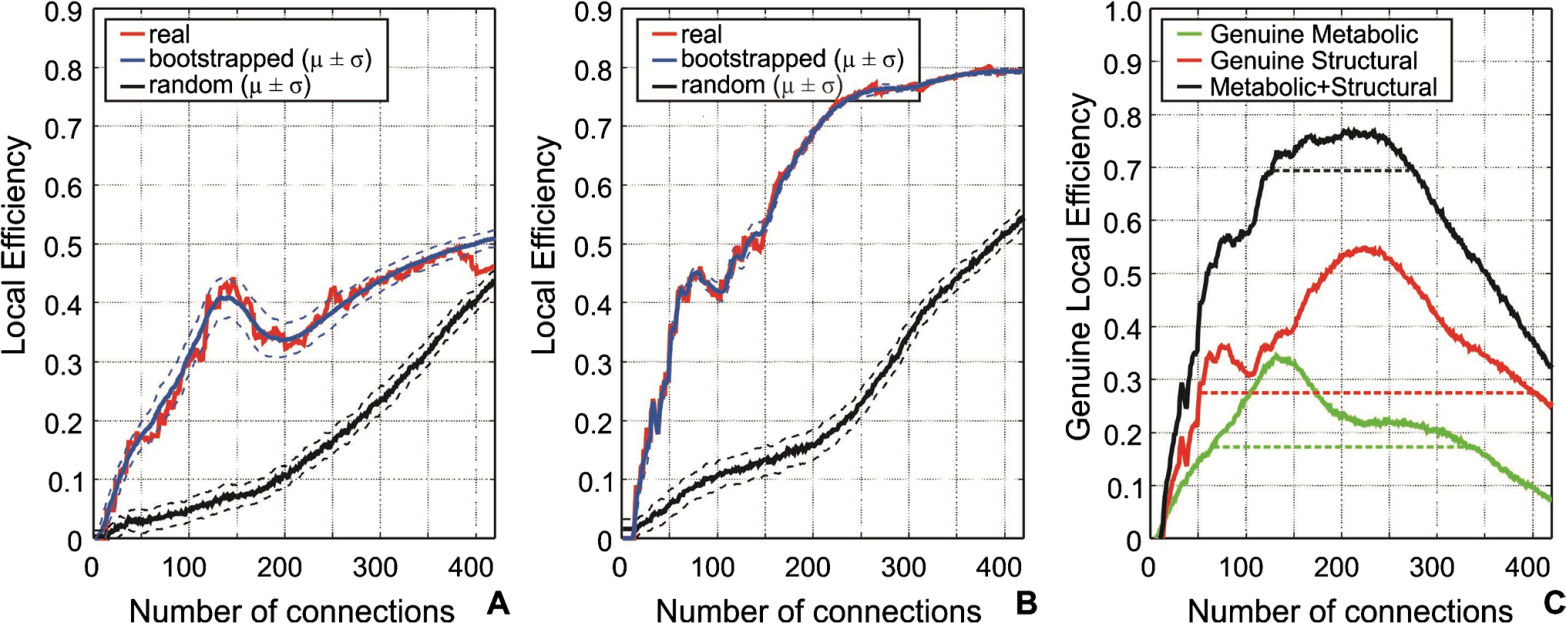
Network efficiency: Local efficiency for FDG_cov_ (**A**) and structural (**B**) connectivity; real data—red line, results of bootstrapping—blue line, and results of randomly generated networks—black line. **C** Genuine local efficiency for FDG_cov_—green line, structural connectivity—red line, and cumulative (summed)—black line. The range of half maximum of FDG_cov_ and structural connectivity networks is illustrated as dashed lines in green and red, respectively. The black dashed line illustrates the 0.9 of maximum total genuine local efficiency, reflecting the optimal number of connections for both networks

**Fig. 3:**
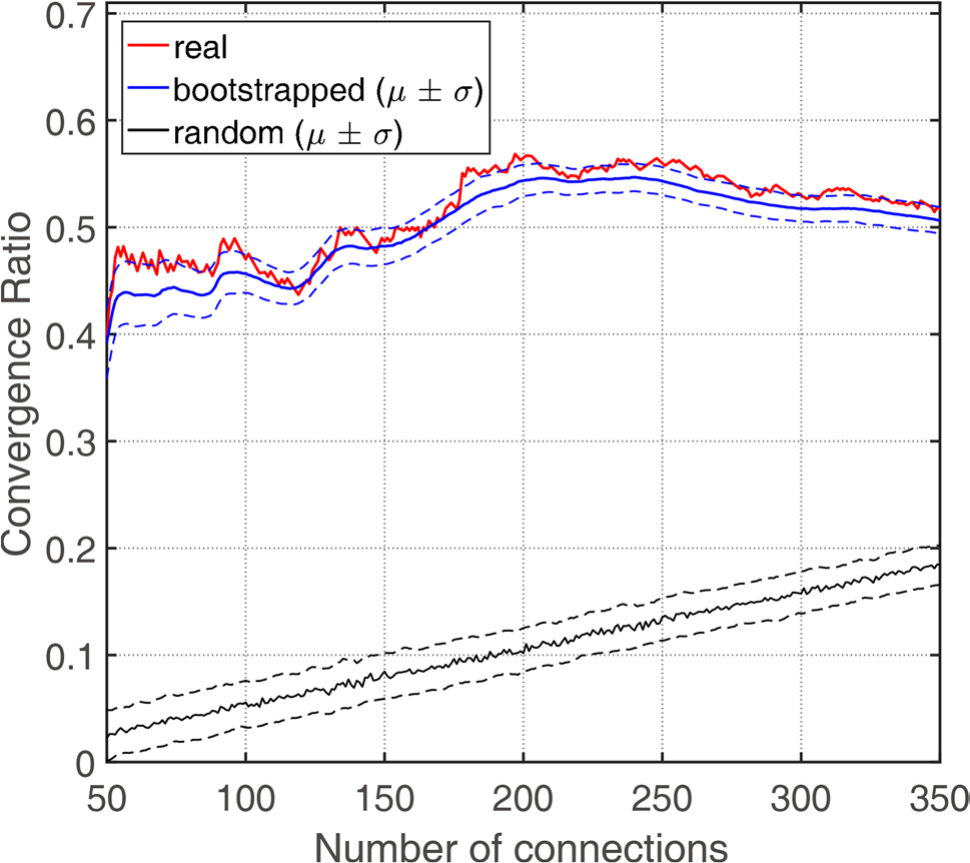
Results of similarity analysis of structural connectivity and FDG_cov_. Values derived from the real data are indicated as red line; bootstrapping results—as blue line and metrics of randomly rewired networks—as black line

Figure 2 shows *LE* of FDG_cov_ and structural networks at different numbers of connections. Both networks appeared to have a significantly higher *LE* compared to the randomly generated networks (FDG_cov_ network: *p* = 1.3 × 10^−178^ and structural network: *p* = 9.4 × 10^−201^). The FDG_cov_ network had a higher variation in the bootstrapped networks (*p* = 2.4 × 10^−174^) as well as lower *LE* (*p* = 2.6 × 10^−158^) than the structural network. In the range of 65–338 connections, both FDG_cov_ and structural networks had a gLE above the half of maximum, such that this range was used for visualization purposes (Fig. 3). A plateau (> 90% of the maximum) of the cumulative gLE of FDG_cov_ and structural connectivity corresponded to the range of connections 128–275 (Fig. 2C). Thus, we used this range in further quantitative analyses.

**Fig. 4:**
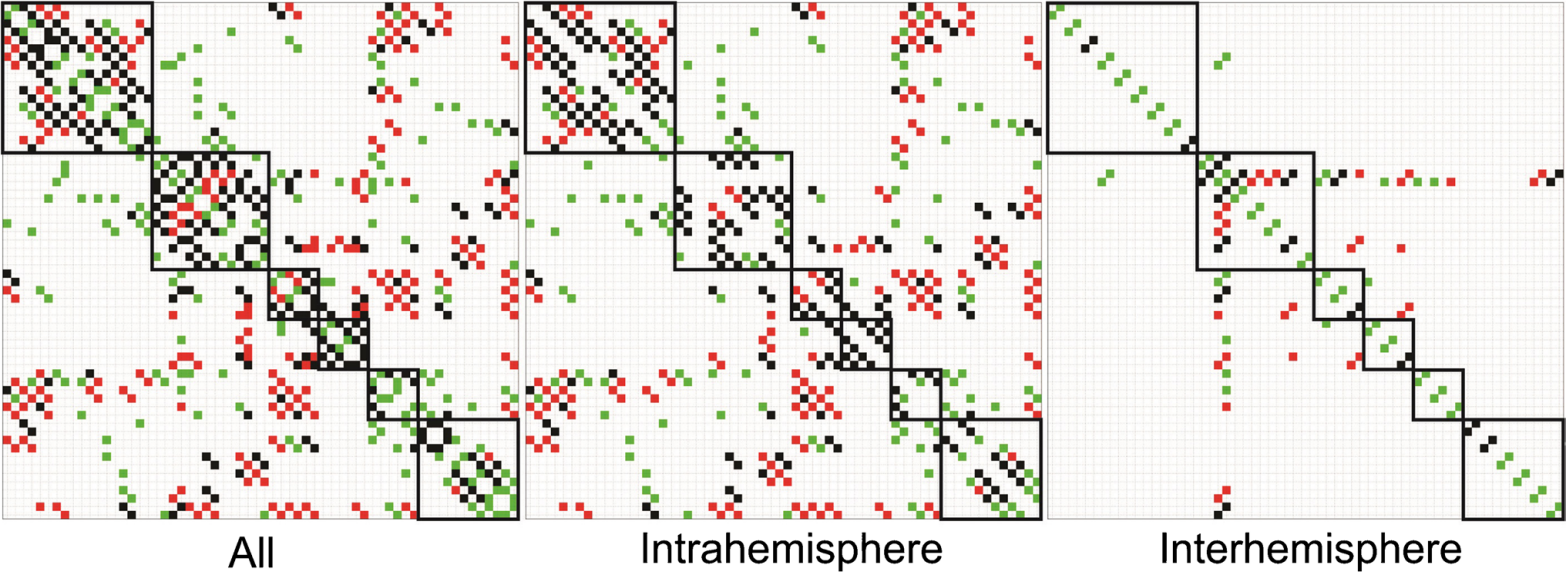
Matrix visualizations of connectivity between 62 regions with 215 connections. FDG_cov_ (green), structural (red), and convergent (black) connections. Boxes capture anatomically related regions, i.e., within (from top to the bottom) the frontal, temporal, parietal, and occipital lobes, subcortical and limbic regions. For regional labels see Supplemental Table 1

**Fig. 5:**
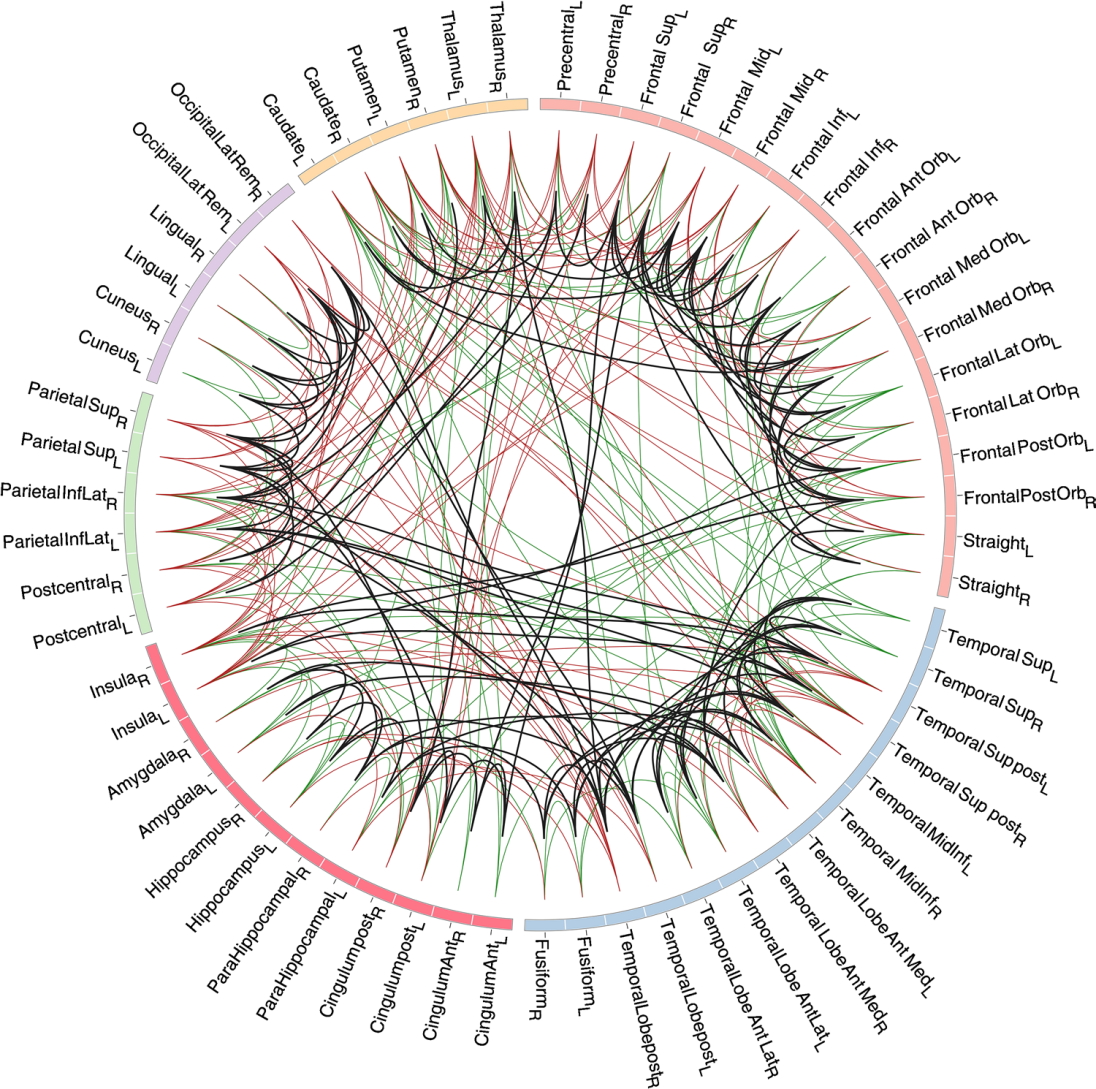
Visualization of FDG_cov_ (green), structural (red), and convergent (black) connections

*CR* of the networks was significantly higher (*p* = 4.9 × 10^−321^) than that by chance (Fig. 3). In the plateau window, i.e., at 128–275 connections, the average *CR* was 0.54 ± 0.03 (0.47–0.57), while *CR* of the random networks was 0.11 ± 0.03 (0.01–0.21).. The results of bootstrapping did not perfectly overlap with the real data results. That is, in Fig. 3, the red line does not exactly follow the solid blue line, likely due to numerical instability of SICE in the process of resampling. Figure 4 depicts matrices of FDG_cov_ and structural networks at ${n}_{connected}=215$, i.e., the maximum of cumulative gLE (Fig. 2C). These networks are depicted in Fig. 5. The proportion of interhemispheric connections was 23% (*n* = 49) and 15% (*n* = 32) in FDG_cov_ and structural networks, respectively. Among intrahemispheric connections, the proportion of intralobe connections was 50% (*n* = 83) and 45.9% (*n* = 84), respectively. Quantitative results for the spatial overlap are summarized in Table 2. *CR* for intralobe and interlobe connections was 0.80 and 0.37, respectively (*p* = 6 × 10^−16^, Wilcoxon rank test). There was no remarkable difference between the hemispheres (Supplemental Table 2). *CR* values for random networks are summarized in Supplemental Table 3. For the whole brain *CR* was 0.12.

**Table 2:** Summary of convergence ratio for 215 connections

	Intrahemisphere	Interhemisphere	Whole brain
All	0.58	–	0.55
Intralobe	0.80	–	–
Interlobe	0.37	0.30	0.36

## Discussion

The present study examined the degree of spatial overlap between FDG_cov_ and structural connectivity. The overlap appeared to be significantly higher for the FDG_cov_ and structural networks than for random ones. At the whole brain level *CR* was 55%. In other words, around a half of detected FDG_cov_ connections in the brain appear to be underlied by white matter tracts. Furthermore, 80% of intralobe FDG_cov_ connections that are supposed to reflect short-range connections [40] were found to have a structural substrate. These results support FDG_cov_ as a sovereign index of brain connectivity.

While a few groups have studied the relationship between fMRI functional and DTI structural connectivity [13, 41, 42], there are still no quantitative data on their spatial overlap. Nevertheless, in line with our data, strong intrahemispheric [43] and weaker transmodal (e.g., interhemispheric) structure–function correlations [44] have been reported. Moreover, a so-called spatial proximity was suggested to be a major determinant of structure–function relationships in diffusion imaging and fMRI data [45]. We found that the proportion of different types of FDG_cov_ connections followed the order of structural ones, i.e., intrahemispheric-intralobe > intrahemispheric-interlobe > interhemispheric-interlobe. These results are in line with DTI studies. In fact, short-range connections were shown to by far outnumber long-range connections passing through corpus callosum [46, 47]. Further, structural connections within an anatomical region were found to be more common than those between regions, followed by interhemispheric connections [48]. However, we detected somewhat more interhemispheric connections in the PET than in the DTI data, i.e., 23% vs. 15%. This is likely related to differences in connectivity modeling. While tractography captures connections underlying an anatomical network of axonal fibers [49], FDG_cov_ modeling “detects” indirect connections if two regions have a similar level of FDG uptake [29, 50]. Given a relative symmetry in cerebral glucose metabolism [51, 52], there is a substantial likelihood that homotopic regions of two hemispheres appear to be connected in FDG-PET data, even though being unconnected anatomically. A similar observation was made by numerous fMRI studies, where strong functional connectivity was found between homotopic regions that are known to be unconnected anatomically (for a review, see Suárez et al. [53]).

While indirect connections in FDG-PET data may be considered as false positive, false negative connections are likely in DTI data. Specifically, deterministic tractography, as used in the present study, terminates streamlines in voxels with sub-threshold FA values. Hence, it was suggested that this tracking approach might miss particularly weak long-range connections (i.e., low FA value at a voxel level) [46, 54]. In the same vein, Sinke et al. reported a relatively high rate of false negative tractography reconstructions for long-range connected cortical areas, as validated against neuronal tracer connectivity measures in the rat [55]. Similarly, post-mortem invasive tracer studies in macaques found that false negatives exhibited a significantly larger connection distance than false positives or true positives [56]. In the present study, the Euclidean distance between any couple of regions of interest from the Hammers atlas negatively correlated with the number of streamlines between the same regions (data not shown), supporting the above notion. Although probabilistic tractography detects so-called kissing fibers in a more sensitive fashion, it seems not advantageous regarding long-range connections [57]. Future tractography studies should test a range of FA thresholds [55].

As a major study limitation, we utilized a heterogeneous cohort that included both neurodegenerative and non-neurodegenerative entities. An unverified assumption behind this pragmatic approach is that a disease equivalently affects FDG_cov_ and structural connectivity. Still, the overlap might differ in the healthy state and in a disease. Further studies should address the impact of data heterogeneity and sample size on estimates of FDG_cov_ in general and on the overlap between FDG_cov_ and structural connectivity in particular. Moreover, a differentiated analysis at the lobe level may produce novel insights into the structural substrates of FDG_cov_.

## Conclusion

Structural connectivity via white matter tracts is a relevant substrate of FDG_cov_, underlying around a half of connections at the whole brain level. Short-range white matter tracts are a major substrate of intralobe FDG_cov_ connections. The present study represents the first valuable reference on structural substrates of FDG_cov_, contributing to establishment of FDG_cov_ as a sovereign index of brain connectome.

## Supplementary Information

Below is the link to the electronic supplementary material.

Supplementary file1 (DOCX 16 KB)

## Data Availability

Data will be made available upon request.

## Code availability

Code will be made available upon request.
