# Supplementary material for: Mapping covariance in brain FDG uptake to structural connectivity
Source: Eur J Nucl Med Mol Imaging. 2021 Oct 22;49(4):1288–97. doi: 10.1007/s00259-021-05590-y (PMC8921091; doi:10.1007/s00259-021-05590-y)
Supplement: Supplementary file 1 — Supplementary file1 (DOCX 16 KB) [file 259_2021_5590_MOESM1_ESM.docx]

**Supplemental table 1.** VOI labels according to the Figure 4

| Frontal lobe | | Temporal lobe | | Parietal lobe | | Subcortical regions | |
| --- | --- | --- | --- | --- | --- | --- | --- |
| 1 | Precentral_L | 19 | Temporal_Sup_L | 33 | Postcentral_L | 45 | Caudate_L |
| 2 | Precentral_R | 20 | Temporal_Sup_R | 34 | Postcentral_R | 46 | Caudate_R |
| 3 | Frontal_Sup_L | 21 | Temporal_Sup_Post_L | 35 | Parietal_InfLat_L | 47 | Putamen_L |
| 4 | Frontal_Sup_R | 22 | Temporal_Sup_Post_R | 36 | Parietal_InfLat_R | 48 | Putamen_R |
| 5 | Frontal_Mid_L | 23 | Temporal_MidInf_L | 37 | Parietal_Sup_L | 49 | Thalamus_L |
| 6 | Frontal_Mid_R | 24 | Temporal_MidInf_R | 38 | Parietal_Sup_R | 50 | Thalamus_R |
| 7 | Frontal_Inf_L | 25 | Temporal_Lobe_Ant_Med_L |  |  |  |  |
| 8 | Frontal_Inf_R | 26 | Temporal_Lobe_Ant_Med_R | Occipital lobe | | Limbic regions | |
| 9 | Frontal_Ant_Orb_L | 27 | Temporal_Lobe_Ant_Lat_L | 39 | Cuneus_L | 51 | Cingulum_Ant_L |
| 10 | Frontal_Ant_Orb_R | 28 | Temporal_Lobe_Ant_Lat_R | 40 | Cuneus_R | 52 | Cingulum_Ant_R |
| 11 | Frontal_Med_Orb_L | 29 | Temporal_Lobe_Post_L | 41 | Lingual_L | 53 | Cingulum_Post_L |
| 12 | Frontal_Med_Orb_R | 30 | Temporal_Lobe_Post_R | 42 | Lingual_R | 54 | Cingulum_Post_R |
| 13 | Frontal_Lat_Orb_L | 31 | Fusiform_L | 43 | Occipital_Lat_Rem_L | 55 | ParaHippocampal_L |
| 14 | Frontal_Lat_Orb_R | 32 | Fusiform_R | 44 | Occipital_Lat_Rem_R | 56 | ParaHippocampal_R |
| 15 | Frontal_Post_Orb_L |  |  |  |  | 57 | Hippocampus_L |
| 16 | Frontal_Post_Orb_R |  |  |  |  | 58 | Hippocampus_R |
| 17 | Straight_L |  |  |  |  | 59 | Amygdala_L |
| 18 | Straight_R |  |  |  |  | 60 | Amygdala_R |
|  |  |  |  |  |  | 61 | Insula_L |
|  |  |  |  |  |  | 62 | Insula_R |

L: left, R: right hemisphere.

**Supplemental table 2.** Summary of convergence ratio for 215 connections separately for the right and left hemisphere

|  | Intra-hemisphere right | Intra-hemisphere left |
| --- | --- | --- |
| All | 0.60 | 0.62 |
| Intralobe | 0.81 | 0.79 |
| Interlobe | 0.38 | 0.37 |

**Supplemental table 3.** Summary of convergence ratio for 215 connections for random networks

|  | Intra-hemisphere | Inter-hemisphere | Whole |
| --- | --- | --- | --- |
| All | 0.14 | - | 0.12 |
| Intralobe | 0.14 | - | - |
| Interlobe | 0.14 | 0.07 | 0.10 |
